# Impact of Chromosome 9 Numerical Imbalances in Oral Squamous Cell Carcinoma: A Pilot Grid-Based Centromere Analysis

**DOI:** 10.3390/diagnostics10070501

**Published:** 2020-07-21

**Authors:** Efthymios Kyrodimos, Aristeidis Chrysovergis, Nicholas Mastronikolis, Evangelos Tsiambas, Christos Riziotis, Dimitrios Roukas, Panagiotis Fotiades, Chara Stavraka, Vasileios Ragos, Minas Paschopoulos, Vasileios Papanikolaou

**Affiliations:** 1 1st ENT Department, Hippocration General Hospital, University of Athens, 115 27 Athens, Greece; timkirodimos@hotmail.com (E.K.); achrysovergis@gmail.com (A.C.); vspap@hotmail.com (V.P.); 2 ENT Department, Medical School, University of Patras, 265 04 Patras, Greece; nmastr@otenet.gr; 3 Department of Cytopathology, 417 Veterans Army Hospital (NIMTS), 115 21 Athens, Greece; 4 Theoretical and Physical Chemistry Institute, Photonics for Nanoapplications Laboratory, National Hellenic Research Foundation, 11635 Athens, Greece; 5 Defence & Security Research Institute, University of Nicosia, CY-2417 Nicosia, Cyprus; 6 Department of Psychiatry, 417 Veterans Army Hospital (NIMTS), 115 21 Athens, Greece; droukas@hotmail.gr; 7 Department of Surgery, 424 General Army Hospital, 564 29 Thessaloniki, Greece; fotias13@hotmail.com; 8 Department of Medical Oncology, Guy’s and St Thomas National Health System Foundation Trust, London SE1 9RT, UK; chara.stavraka@gmail.com; 9 Department of Maxillofacial Surgery, Medical School, University of Ioannina, 455 00 Ioannina, Greece; vasragkos@gmail.com; 10 Department of Obstetrics and Gynaecology, School of Health Sciences, University of Ioannina, 455 00 Ioannina, Greece; mpasxop@uoi.gr

**Keywords:** carcinoma, oral, chromosome, polysomy, grid, microscopy

## Abstract

Oral squamous cell carcinoma (OSCC) is considered an aggressive malignancy, mainly due to its increased propensity to provide local and distant lymph node metastases. Gross chromosome instability (CI; polysomy/aneuploidy/monosomy), combined or not with specific gene alterations, is implicated in the development and progression of solid malignancies, including OSCC. In order to further study the relationship between these genetic alterations and the aggressive biological behavior of OSCCs, we investigated the frequency and impact of chromosome 9 numerical imbalances in these tumors. Fifty (*n* = 50) formalin-fixed, paraffin-embedded primary OSCC tissue sections were used. Chromogenic in situ hybridization (CISH) was implemented for detecting chromosome 9 (CEN—centromere enumeration) numerical alterations. Concerning the screening process in CISH slides, a novel, real-time reference and calibration grid platform was implemented. Chromosome 9 polysomy was observed in 8/50 (16%) tissue sections, whereas the rest of them demonstrated a normal, diploid pattern (42/50; 84%). Chromosome 9 polysomy was associated with the grade of differentiation of the examined tumors (*p* = 0.036). Chromosome 9 numerical imbalances (polysomy) were observed in sub-groups of OSCCs correlating with a progressive dedifferentiation of the malignant tissues. Concerning the implementation of the proposed grid-based platform as described above on CISH slides, it provides a novel, fast, and accurate screening mapping mechanism for detecting chromosome numerical imbalances.

## 1. Introduction

Development and implementation of modern molecular biology techniques has led to the detection of a broad spectrum of genomic imbalances in solid malignancies. Various gross chromosome instabilities (CIs), such as polysomy/aneuploidy/aneusomy, either combined or not with specific gene deregulation mechanisms, including point mutations, deletions, and amplification, have been noted in these tumors. These critical genetic events have been associated with alterations of the prognosis and the response rates to novel targeted therapeutic regimens in the corresponding patients [1]. In the oral cavity, oral squamous cell carcinoma (OSCC) is the most frequent type of malignancy. Interestingly, this pathological entity is frequently characterized by an aggressive phenotype due to an increased tendency to present local and distant lymph node metastases, apparently as a result of severe genetic alterations [2]. Various etiopathogenetic factors have been associated with OSCC development and progression including tobacco, chronic alcohol consumption, and viral-mediated deregulation [3,4]. Concerning viral oncogenic activity, persistent human papillomavirus (HPV) infection is responsible for malignant transformation of the affected oral/oropharyngeal epithelia modifying the host cell genome [5]. Chromosome 9 consists of approximately 138 million base pairs of nucleic acids referring to 4.5 percent of the total DNA in cells. Significant genes such as endoglin (9q34.11), caspase recruitment domain (9q22.31), transforming growth factor beta, receptor (9q22.23), and, of course, the p16^INK4a^ suppressor gene (9p21) are hosted on its p and q arms [6]. In OSCC, chromosome 9 instability is under investigation regarding its impact on the corresponding patients’ clinicopathological features. In the current study, we analyzed chromosome 9 numerical status in OSCC tissues by implementing a chromogenic in situ hybridization (CISH) assay in order to identify sub-groups of patients with specific CI patterns, applying a novel, grid-based coverslip platform for a systematic and accurate CISH slide screening process.

## 2. Results

According to CISH implementation and bright-field centromere signal grid-based analysis, chromosome 9 polysomy was observed in 8/50 (16%) tissue sections, whereas the rest demonstrated a normal, diploid pattern (42/50; 84%). Chromosome 9 polysomy was associated with the grade of differentiation of the examined tumors (*p* = 0.036). In fact, polysomy was observed in grade 2/3 cases, reflecting an association with a progressive dedifferentiation of the malignant tissues. No further statistical correlations were identified regarding the other clinical–pathological parameters (gender: *p* = 0.572; stage: *p* = 0.841; smoking status: *p* = 0.661; persistent HPV infection: *p* = 0.923). Additionally, there was no significant correlation between chromosome 9 numerical imbalances and age of the examined patients (*p* = 0.201). Concerning the impact of this novel, grid-based, real-time method, we observed that it provided improved, rapid whole-slide-surface eye-scanning and mapping compared to the conventional eye microscopy procedure. In fact, grid-based, systematic screening detected a remarkably increased number of nuclei characterized by centromere 9 numerical imbalances in the examined stained slides (mean value 39 vs. 22, respectively; *p* = 0.029), leading to a more accurate CISH score, especially in borderline cases.

## 3. Discussion

CI comprises polysomy/aneuploidy (usually 3–6 chromosome copies per nucleus) and monosomy (loss of one chromosome) even rearrangements (i.e., translocations) detectable by karyotyping and in situ hybridization techniques. Implementation of conventional fluorescence in situ hybridization (FISH)-based assays or, alternatively, CISH analyses are the optimal molecular tools for gene and chromosome numerical imbalance evaluation in solid malignancies [7,8,9]. Concerning CI identification in OSCC and in pre-malignant lesions, such as oral lichen planus, comparative molecular studies have shown that there are differences regarding the chromosomes that demonstrate aneuploidy in sub-groups of patients, affecting also OSCC local recurrence [10,11]. Focused on chromosomes 9, 8, 11, and 17, a study group concluded that there is a significant intra-tumoral chromosomal heterogeneity in these malignancies [12].

In the current study, we analyzed chromosome 9 numerical status in OSCC tissues in order to identify sub-groups of patients with specific CI patterns. We focused on this specific chromosome due to the limited published data regarding OSCC, especially analyzing its numerical imbalances by CISH molecular technique. Furthermore, the chromosome 9 hosts very important genes, including p16^ink4^ (gene band: 9p21), a crucial suppressor gene that is frequently deregulated in solid malignancies, including OSCC. We detected chromosome 9 polysomy in a sub-group of the examined specimens with specific pathological characteristics. We observed that polysomy was observed in grade 2/3 cases, reflecting an association with a progressive dedifferentiation of the malignant tissues. Furthermore, in high risk (HR)-HPV cases, the described genetic instability was also obvious, although overall analysis did not provide statistical significance. Another FISH-based analysis on cytological substrates (fine-needle aspirates) detected elevated CI regarding chromosomes 7, 9, and 11, correlated with reduced disease-free survival and overall survival in the corresponding patients [13]. Similar studies have shown that specific chromosome imbalances, including 9q, 8q, and 11q gains and 3p and 4q losses, are correlated with poor prognosis [14]. Furthermore, loss of heterozygosity (LOH) seems to be a crucial genetic alteration in the development and progression of OSCC, including 9p locus. Using polymorphic microsatellite markers such as D9S1748 at 9p21 or D9s1747, RPS6, or D9s162, various studies detected increased levels of LOH, especially in chronic mucosal trauma substrates (inflammatory fibrous hyperplasia) associated with OSCC development and high rates of local recurrences, respectively [15,16]. Some molecular studies analyzing genomic imbalances at chromosome 9p concluded that 9p21 silencing—due to LOH combined or not with methylation of the p16 gene-promoter region—and also 9p13 gain seem to be involved in the development and progression of OSCCs as early genetic alterations [17,18,19,20]. Interestingly, the combination of specific chromosome 3 and 9 loci (3p14 and 9p21, respectively) losses combined with polysomy was assessed by a study group applying a FISH assay [21]. They observed that in oral leukoplakias, these gene and gross chromosome alterations are critical for the progression of the lesions to in situ carcinoma, whereas polysomy 3 was detected more frequently than polysomy 9. Concerning the impact of chromosome 9 in the corresponding patients based on their age, our study showed no relation. Some studies have reported a borderline correlation between younger patients and specific genetic markers—combined or not with smoking status and HR-HPV persistent infection—in OSCC development and progression, but others support controversial results with no differences when comparing young to older patients [22,23,24]. In the current experimental study, we applied an innovative reference and calibration grid on conventional coverslips as a pilot screening mechanism for CISH slide evaluation. We have already reported this improved technique as a tool for systematic screening in immunocytochemistry-stained slides [25]. It is an easy-to-use, accurate tool for eye-based slide scanning under bright-field microscopy with a broad spectrum of applications including cytological (conventional/liquid-based) and molecular (CISH) slides.

## 4. Materials and Methods

### 4.1. Study Group

For the purposes of our study, fifty (*n* = 50) archival, formalin-fixed, and paraffin-embedded tissue specimens of histologically confirmed primary OSCCs were used. It should be mentioned that all the corresponding histopathological material was derived from surgical operations at the beginning of the diagnosis of the disease as a primary malignancy and not from a local recurrence. The hospital ethics committee consented to the use of these tissues in the Department of Pathology (Reference ID research protocol: 2226, approved on 9 September 2018), Hippocration Hospital, University of Athens, Athens, Greece, for research purposes, according to World Medical Association Declaration of Helsinki guidelines (2008, revised 2014). The tissue samples were fixed in 10% neutral-buffered formalin. Hematoxylin and eosin (H&E)-stained slides of the corresponding samples were reviewed for confirmation of the histopathological diagnoses. All lesions were classified according to the histological typing criteria of the World Health Organization (WHO) Pathology Series [26]. The majority of the examined lesions were derived from the tongue (*n* = 46), whereas the rest of them (*n* = 4) were derived from the retromolar trigone (1), the floor of the mouth (2), and the buccal mucosa (1). Staging classification was based on the American Joint Committee Cancer (AJCC) seventh edition (AJCC7) in the current retrospective study. Concerning HPV DNA status (positivity or not), the corresponding information was derived from patients’ medical file records. Among them, 18 HPV DNA-positive cases were recorded. HPV 16/31/53 high-risk (HR) subtypes were detected mainly by analyzing the corresponding cases. In this randomized study, there were no other strict inclusion or exclusion criteria regarding the selection of the OSCC specimens and the corresponding patients. Clinicopathological data of the examined cases are demonstrated in Table 1.

### 4.2. Chromogenic In Situ Hybridization (CISH) Assay

**Figure 1:**
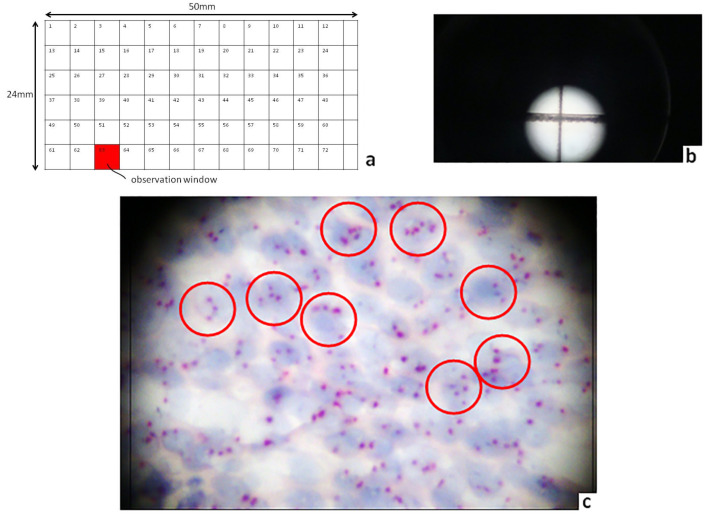
Chromosome 9 CISH analysis in OSCC. (**a**) Schematic presentation of the grid-based coverslip that was implemented in CISH slide screening mapping. Note this prototype grid consisting of seventy-two (*n* = 72) rectangular square areas arranged in 12 columns and 6 rows. The square numbered 63 simply indicates characterically an individual observation window (**b**) A snapshot of the grid-based coverslip demonstrating a fine cross made by laser inscription that splits its surface into four square segments. (**c**) Chromosome 9 polysomy in a malignant tissue section. Note 3 to 5 isolated, light-brown centromeric signals per nucleus in a sub-group of the stained nuclei (inside red circles DAB chromogen; original magnification 400×).

For the purposes of our study, we selected and applied the SPOT LIGHT CISH assay (Zymed/InVitrogen, San Francisco, CA, USA) based on centromere chromosome enumeration probe 9 (CEN 9). In brief, the sections were deparaffinized and incubated in pre-treatment buffer in a temperature-controlled microwave oven at 92 °C for 10 min using a Spot-Light formalin-fixed, paraffin-embedded reagent kit (Zymed Inc., San Francisco, CA, USA). The sections were then allowed to cool for 20 min and then washed with phosphate-buffered saline. Enzymatic digestion was carried out by applying 100 mL of formalin-fixed, paraffin-embedded digestion enzyme onto the slides for 10 to 15 min at room temperature. The slides were washed with phosphate-buffered saline and dehydrated with graded ethanol. The ready-to-use biotin-labeled probe was applied onto the slides, which were covered with a 14 × 14 mm coverslip. The slides were denatured on a hot plate at 94 °C for 3 min, and hybridization was performed overnight at 37 °C. After hybridization, the slides were washed with 0.5% standard saline citrate for 5 min at 75 °C, followed by 3 washes in phosphate-buffered saline/0.2% Tween 20 at room temperature. The probe was detected with sequential incubations with avidin–peroxidase and 3,3-diaminobenzidine (DAB) according to the manufacturer’s instructions (Zymed Inc., San Francisco, CA, USA). Tissue sections were lightly counterstained with hematoxylin, dehydrated in graded ethanol, and embedded. At the end of the process, CISH Chromosome Enumeration Probe (CEP) 9 centromeric signals were easily visualized as dark-brown, scattered dots using a conventional bright-field microscope (Figure 1). Interpretation of the chromosome 9 centromeric signal was based on Zymed’s evaluation chart for CISH. According to this guide, two centromere copies per nucleus demonstrate a normal, diploid pattern, whereas three and more centromere copies per nucleus characterize chromosome polysomy in >= 50% or small clusters of the examined intact, non-overlapped cancerous nuclei on every stained slide. Additionally, one centromeric signal per nucleus represents chromosome monosomy (loss of one chromosome).

### 4.3. Slide Screening Process

The screening procedure regarding the corresponding archived CISH-stained slides was performed using bright-field microscopy (microscope Olympus CX-31, Menvile, NY, USA) with ToupView image analysis software (ProWay/ToupTekProtonics, Hangzhou, China) with combined 100×/400× magnification. Concerning the CISH slide evaluation, screening was based on a set of novel designed coverslips with an integrated spatial rectangular grid (Grid Cover Slip, now GCS) in order to provide an efficient way of slide eye-scanning, ensuring a systematic inspection with full visual coverage of the slide. The grid patterns on GCSs were produced by applying a prototype, home-built, high-precision, and efficient femtosecond-laser-based micromachining system (Femtosecond Laser Micromachining—FLM) based on a high-power femtosecond fiber laser (Model: HE-1060-1μJ-fs, Fianium, Southampton, UK). Laser inscription techniques can allow direct writing and transfer of predetermined patterns by means of surface or sub-surface micromachining in a variety of materials ranging from glasses to soft polymeric materials. Adjusting the laser writing characteristics, the inscribed visible grid lines’ width could be flexibly defined to a range, typically from 1 μm to 500 μm. The FLM inscription technique has been applied here for the first time toward the fabrication of a visible, rectangular grid in a microscope CISH slide’s coverslip for research purposes. Commercially available coverslips made of typical borosilicate-based glass, 50 × 24 × 0.5 mm (length × width × thick) (Menarini, Florence, Italy), were used in the study. The grid’s size and observation windows’ density could be modified according to the researcher’s needs. Based on our previous experience in Pap test slide screening-mapping, we selected a prototype grid consisting of seventy-two (*n* = 72) rectangular squares of a typical surface area of 4 × 4 mm, equal to 16 mm^2^, arranged in 12 columns and 6 rows [27]. Each square segment can have also appropriate spatial-indexing marks (printed with various techniques like FLM) in a suitable way to assure minimum visual interference under microscope inspection (Figure 1b). The indexing can be provided by sequential numbering of the square cells as shown schematically in Figure 1a. In the specific example, due to the selection of the grid’s window size, six residual, non-rectangular cells, numbered in the figure as 73–78, appear in the right part of the grid and do not affect the generalization of the proposed grid’s architecture. The developed GCSs were used for the visual detection of Chr 9 centromeric signals on the corresponding CISH slides by perfectly covering the entire conventional coverslip. At the end of the microscopic process, both (conventional and novel grid-based) methods were compared on the basis of total centromere 9 copy detection in CISH slides.

### 4.4. Statistical Analysis

Statistics software package IBM SPSS v25 (SPSS Inc., Chicago, IL, USA) was implemented. Associations between variables were assessed with the Pearson chi-squared (χ^2^) test and Fisher’s exact test. Correlation analysis with the Spearman rank test was performed for variables with significant chi-squared associations. Two-tailed *p* values ≤ 0.05 were considered statistically significant. Results and correlations (*p* values) are described in Table 1.

## 5. Conclusions

Our study showed that chromosome 9 numerical imbalances (polysomy) are observed in sub-groups of OSCCs correlating with a progressive dedifferentiation of the malignant tissues. Concerning the implementation of the proposed grid-based platform on CISH slides as described above, it provides a novel, fast, and accurate screening–mapping mechanism for detecting chromosome numerical imbalances.

## 6. Patents

Riziotis, C.; Tsiambas, E. Reference and Calibration Grid for Medical Diagnostic Microscopy. Patent HIPO/OBI GR #10008931, 16 July 2015; Patent Application PCT/GR2016/000032, 15 July 2016; Patent Application WO2017/009673, 19 January 2017.

## Figures and Tables

**Table 1:** Clinicopathological parameters and total chromogenic in situ hybridization (CISH) chromosome 9 results.

Clinicopathological Parameters		Chromosome 9 (CEN)		*p* Value
OSCC (*n* = 50)		N/D	P	
		42/50 (84%)	8/50 (16%)	
	*n* (%)	*n*	*n*	
**Gender**				0.572
Male (mean age: 54.9)	44 (88%)	36/50 (72%)	8/50 (16%)	
Female (mean age: 49.1)	6 (12%)	6/50 (12%)	0/50 (0%)	
**HPV history**				0.923
Positive	18 (36%)	15/50 (30%)	3/50 (6%)	
Negative	32 (64%)	27/50 (54%)	5/50 (10%)	
**Grade**				0.036
1	18 (18%)	18/50 (36%)	0/50 (0%)	
2	21 (58%)	19/50 (38%)	2/50 (4%)	
3	11 (24%)	3/50 (6%)	6/50 (12%)	
**Stage**				0.841
I	9 (18%)	9/50 (18%)	0/50 (0%)	
II	26 (52%)	22/50 (44%)	4/50 (8%)	
III	15 (30%)	11/50 (22%)	4/50 (8%)	
**Smoking status**				0.661
Current	38 (76%)	31/50 (62%)	7/50 (14%)	
Former	12 (24%)	11/50 (22%)	1/50 (2%)	

OSCC: oral squamous cell carcinomas, N/D: normal/diploid pattern, P: polysomy, CEN: centromere enumeration.

## Abbreviations

AJCC7	American Joint Committee Cancer seventh edition
OSCC	Oral squamous cell carcinoma
CI	Chromosome instability
CISH	Chromogenic in situ hybridization
CEN	Centromere enumeration
LOH	Loss of heterozygosity
WHO	World Health Organization
GCS	Grid coverslip
FLM	Femtosecond laser micromachining
DAB	3,3-diaminobenzidine
